# FG-DeformT: Grouped Deformable Temporal Modeling for Flight-Level Aircraft Anomaly Detection

**DOI:** 10.3390/s26103189

**Published:** 2026-05-18

**Authors:** Yinpan He, Zhen Lu, Yi Deng, Di Wang

**Affiliations:** 1 Faculty of Data Science, City University of Macau, Macao 999078, China; d230921102441@cityu.edu.mo (Y.H.); d23092110441@cityu.edu.mo (Y.D.); d23092110208@cityu.edu.mo (D.W.); 2 School of Computer Engineering, Guangzhou City University of Technology, Guangzhou 510800, China

**Keywords:** aircraft anomaly detection, multivariate time series, Transformer, flight-level validation, cross-flight generalization, fault isolation

## Abstract

Aircraft anomaly detection from multivariate telemetry time series is critical for flight safety and predictive maintenance. However, random window-level evaluation may overestimate performance because highly correlated windows from the same flight can appear in both the training and test sets. To obtain a stricter assessment of cross-flight generalization, this study adopts a leakage-aware flight-level validation protocol and proposes FG-DeformT, a grouped deformable Transformer for aircraft time-series anomaly detection. The model combines global temporal modeling with a grouped temporal offset mechanism to capture heterogeneous local temporal shifts across latent feature subspaces. Experiments on the ALFA dataset show that conventional baselines degrade sharply under flight-level evaluation, whereas FG-DeformT remains robust, achieving a Precision of 0.925, a Recall of 0.941, and an F1-score of 0.933 on the primary binary anomaly detection task. Ablation and downstream analyses further indicate that the grouped offset mechanism contributes to anomaly detection, while its benefit for fine-grained fault isolation is category-dependent rather than uniformly superior. External validation and computational analyses are included to further examine the framework’s applicability and practical feasibility. Overall, this study highlights the importance of leakage-aware flight-level evaluation and suggests that grouped temporal deformation offers a practical way to model heterogeneous temporal patterns in safety-critical telemetry data.

## 1. Introduction

Aircraft systems generate large volumes of multivariate sensor time-series data during flight, recording engine conditions, control inputs, and system states. These data provide the basis for onboard health monitoring and post-flight fault analysis. Timely anomaly detection is therefore important for improving maintenance efficiency, preventing severe failures, and enhancing flight safety [1,2,3,4].

Recent advances in deep learning have substantially improved data-driven fault diagnosis and anomaly detection. Earlier studies commonly relied on recurrent or convolutional architectures, such as LSTM-, CNN-, and BiLSTM-based models, to capture nonlinear temporal patterns in aviation data [5,6,7]. More recently, Transformer-based models have attracted increasing attention because their self-attention mechanism improves the modeling of long-range temporal dependencies and complex inter-variable interactions [8,9,10]. These developments have expanded the methodological toolbox for multivariate time-series anomaly detection.

However, an important evaluation issue remains insufficiently addressed. Most studies have adopted random window-level splitting, in which sliding windows sampled from the same flight may appear in both the training and test sets. Because neighboring windows from one flight usually share similar operating conditions, environmental context, and aircraft states, such splits can introduce strong correlation between training and testing samples. As a result, high reported performance may partly reflect flight-specific memorization rather than genuine transfer to unseen flights [11,12]. This issue is especially problematic for aircraft anomaly detection, where practical deployment requires robust generalization across different flights.

To better reflect this scenario, we adopt a flight-level evaluation protocol, in which all samples from the same flight are assigned to the same partition. This setting provides a more realistic assessment of cross-flight generalization and exposes the difficulty of anomaly detection under distribution shifts across flight trajectories.

Under this protocol, we propose a flight-generalizable grouped deformable Transformer (FG-DeformT) for aircraft multivariate time-series anomaly detection. The model combines Transformer-based global temporal modeling with a grouped temporal offset branch that captures heterogeneous local temporal variations from different feature subspaces. This design aims to improve anomaly-sensitive representation learning under cross-flight distribution shifts.

The main contributions of this paper are as follows:

- A leakage-aware flight-level evaluation framework is adopted for aircraft multivariate time-series anomaly detection. Instead of randomly splitting highly correlated sliding windows, all samples from the same flight are kept within the same partition. This setting provides a stricter and more realistic assessment of cross-flight generalization.
- A grouped deformable temporal modeling mechanism is introduced to capture heterogeneous temporal shifts in aircraft telemetry. The proposed FG-DeformT partitions latent representations into feature groups and estimates group-specific temporal offsets, enabling the model to represent asynchronous local temporal variations that may be difficult to capture with standard self-attention alone.
- Comprehensive experiments are conducted under both random window-level and flight-level evaluation settings. The results show that conventional baselines can achieve inflated performance under random splitting but degrade substantially under flight-level evaluation, whereas FG-DeformT maintains strong performance on the primary binary anomaly detection task.
- Extended ablation and boundary analyses are performed to clarify the role and limitation of the grouped temporal offset mechanism. The results indicate that the proposed mechanism is effective for flight-level anomaly detection, while its benefit for downstream fine-grained fault isolation is category-dependent rather than universally superior.

## 2. Related Work

### 2.1. Aircraft Fault Diagnosis and Anomaly Detection

Aircraft fault diagnosis has gradually shifted from rule-based and shallow learning methods to data-driven temporal modeling. Recent studies have applied recurrent, convolutional, and hybrid neural architectures to aviation monitoring and diagnosis tasks, showing the effectiveness of learning fault patterns directly from sensor time series [13,14,15]. However, generalization across unseen flights remains insufficiently studied, despite being critical for realistic deployment [16,17].

### 2.2. Transformer-Based Multivariate Time-Series Modeling

Transformer-based models have recently become an important direction for multivariate time-series anomaly detection, as self-attention can capture long-range temporal dependencies and inter-variable interactions. Existing work has proposed a variety of Transformer-based designs for anomaly detection and representation learning [9,10,18,19]. However, most studies have performed evaluation under random window-level splitting, which may not reflect generalization to unseen aircraft flights.

### 2.3. Evaluation Protocols and Generalization

Sliding-window samples are often strongly correlated rather than independent. When windows from the same trajectory are randomly assigned to different partitions, evaluation may suffer from leakage and yield overly optimistic results [11,12,20]. This motivates flight-level partitioning as a stricter and more realistic protocol for aircraft anomaly detection.

Despite these advancements, a critical gap remains: the discrepancy between laboratory evaluation accuracy and real-world deployment reliability. Most existing literature focuses on refining model architectures under the assumption of independent and identically distributed samples, neglecting the temporal continuity and flight-specific context inherent in aircraft telemetry. This oversight motivates us to reconsider the fundamental problem formulation for aircraft anomaly detection.

## 3. Problem Formulation

### 3.1. Definition of Anomaly Detection Task

In light of the limitations identified in existing work, we reformulate aircraft anomaly detection not merely as a sequence classification task, but as a cross-flight generalization problem. Let $X\in R^{T\times D}$ denote an input time-series window, where *T* is the sequence length, and *D* is the number of sensor variables. Each sample is associated with a binary label y∈{0,1}, where y=0 denotes a normal state, and y=1 denotes an anomalous or fault state. The objective is to learn a mapping f(X)→y that can accurately distinguish anomalous flight segments from normal ones.

Compared with conventional classification tasks, anomaly detection generally faces challenges such as sparse abnormal patterns, weak fault signatures, and strong dependence on temporal context. In aircraft telemetry, these difficulties are further compounded by cross-flight distribution shifts caused by differences in environmental conditions, operating states, equipment status, and flight-specific dynamics. Therefore, an effective detector should not only capture discriminative temporal patterns within each window, but also generalize well to unseen flights with potentially different data distributions.

### 3.2. Flight-Level Validation Protocol

A central concern of this work is the evaluation protocol. In many studies, sliding windows extracted from the same time series are randomly partitioned into training and testing sets. Although such a random-window-level protocol is convenient, it may lead to overly optimistic performance estimates when strongly correlated samples from the same flight appear in both splits. The fundamental issue is that adjacent windows within the same flight are not statistically independent, since they share the same operational context, environmental conditions, and aircraft state. This data leakage problem obscures the model’s true generalization capability in real-world scenarios.

**Figure 1:**
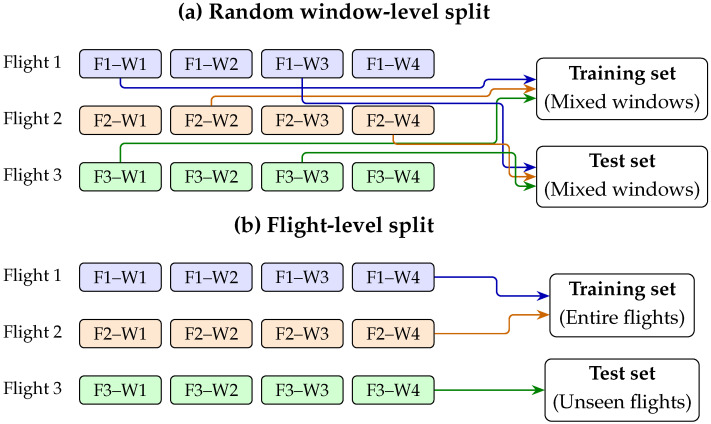
Comparison between random window-level and flight-level validation protocols. Random splitting may mix highly correlated windows from the same flight across training and test sets, whereas flight-level splitting keeps each flight within a single partition and evaluates the model on unseen flights. Different colored arrows indicate windows originating from different flights.

To better reflect realistic deployment conditions, we adopt a flight-level validation protocol. In this setting, all samples from the same flight are kept in the same partition, ensuring that the model is evaluated on flights that were completely unseen during training. This protocol prevents flight-specific information leakage and provides a stricter test of cross-flight generalization ability [12,20]. Figure 1 illustrates the difference between the conventional random window-level split and the flight-level split adopted in this study.

For comparison, we also report results under a random window-level protocol. The difference between these two evaluation settings allows us to examine how much conventional random splitting may inflate the apparent performance of anomaly detection models, thereby highlighting the necessity of the flight-level protocol. However, random-split results are used only as a diagnostic reference, whereas flight-level grouped validation is used as the primary basis for model comparison.

### 3.3. Extended Fault Isolation Task

Beyond the primary anomaly detection task, we further consider an extended fault isolation task as a downstream analysis. In this setting, samples identified as anomalous are further categorized into specific fault types. Let $y_{f}\in \left\{1,2,\ldots ,C\right\}$ denote the fault category label, where *C* is the number of valid fault classes after excluding the normal class.

The purpose of this extended task is not to replace the main anomaly detection objective but to provide a more detailed analysis of whether the learned temporal representations are informative enough for fine-grained fault discrimination. This task is therefore treated as a complementary evaluation rather than the primary target of the proposed framework.

### 3.4. Evaluation Metrics

For the main anomaly detection task, we adopt Precision, Recall, and F1-score as the primary evaluation metrics. These metrics are defined as

(1) $$\mathrm{Precision}=\frac{TP}{TP+FP},$$

(2) $$\mathrm{Recall}=\frac{TP}{TP+FN},$$

(3) $$F1=\frac{2\cdot \mathrm{Precision}\cdot \mathrm{Recall}}{\mathrm{Precision}+\mathrm{Recall}},$$

where TP, FP, and FN denote the numbers of true positives, false positives, and false negatives, respectively.

For the extended fault isolation task, we additionally report Accuracy, Weighted F1, and Macro F1. Accuracy reflects the overall proportion of correctly classified samples. Weighted F1 emphasizes global performance under imbalanced class distributions, while Macro F1 treats each class equally and is therefore more suitable for evaluating performance balance across different fault categories.

By jointly considering these metrics, we are able to comprehensively assess a model’s anomaly detection performance, robustness, and class-level behavior under the flight-level evaluation protocol.

## 4. Methodology

### 4.1. Overall Architecture of FG-DeformT

**Figure 2:**
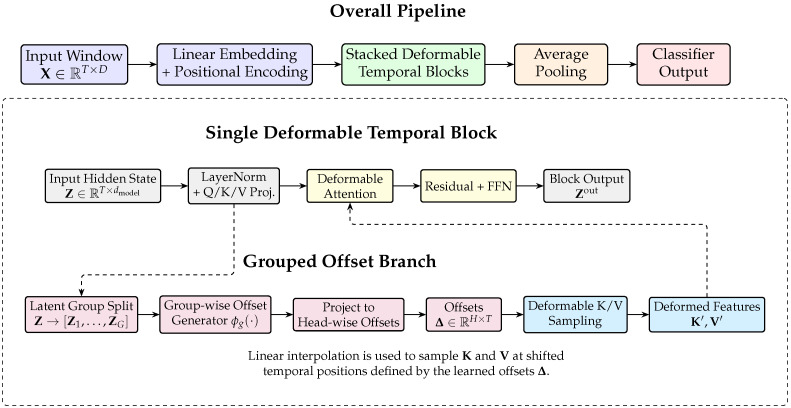
Overall architecture of FG-DeformT. The model first embeds the multivariate input window and processes it with stacked deformable temporal blocks. Within each block, the hidden representation is divided into latent groups, and group-wise offset generators estimate temporal displacements. The learned offsets are projected to head-wise offsets and used to deform the sampling positions of the key and value representations, yielding deformed features $K^{\prime }$ and $V^{\prime }$ for attention computation. The final representation is aggregated by average pooling and mapped to the anomaly or fault label.

FG-DeformT is designed for flight-level generalizable anomaly detection from aircraft multivariate telemetry time series. Figure 2 illustrates the overall architecture of FG-DeformT. Given an input multivariate telemetry window, the model first applies linear embedding and positional encoding, and then processes the sequence using stacked deformable temporal blocks. Within each block, the latent representation is partitioned into feature groups, and group-specific offset generators estimate temporal displacements. These offsets are projected to head-wise temporal offsets and used to deform the sampling positions of the key and value representations before attention computation. In this way, the model preserves the global dependency modeling capability of self-attention while explicitly accounting for local asynchronous temporal variations. The final sequence representation is aggregated and mapped to the anomaly or fault label.

The flight-level partitioning protocol is not part of the model architecture. Instead, it is used as a leakage-aware evaluation setting to assess whether the learned representations generalize to unseen flights. The model itself focuses on learning anomaly-sensitive temporal representations under this stricter setting.

### 4.2. Input Embedding and Latent Feature Grouping

The raw input window is first mapped into a latent space:

(4) $$H^{\left(0\right)}=XW_{e}+b_{e},$$

where $W_{e}\in R^{D\times d_{\mathrm{model}}}$ and $b_{e}\in R^{d_{\mathrm{model}}}$ are learnable parameters. A learnable positional encoding $P\in R^{T\times d_{\mathrm{model}}}$ is then added:

(5) $$Z^{\left(0\right)}=H^{\left(0\right)}+P.$$

Within each deformable temporal block, the normalized hidden representation is denoted as $Z\in R^{T\times d_{\mathrm{model}}}$. To model heterogeneous temporal responses, the latent feature dimension is divided into *G* non-overlapping groups:

(6) $$Z=[Z_{1},Z_{2},\ldots ,Z_{G}],$$

where $Z_{g}\in R^{T\times d_{g}}$ denotes the representation of the *g*-th group and $\sum_{g=1}^{G}d_{g}=d_{\mathrm{model}}$. In our implementation, groups are formed by evenly splitting the latent feature channels rather than manually assigning physical sensor categories. When $d_{\mathrm{model}}$ is divisible by *G*, $d_{g}=d_{\mathrm{model}}/G$; otherwise, the remaining channels are assigned to the last group. This grouping strategy keeps the module simple and reproducible while allowing different latent subspaces to learn different temporal deformation patterns.

### 4.3. Group-Specific Temporal Offset Generation

The key component of FG-DeformT is the group-specific temporal offset generation branch, which is designed to model local temporal misalignment among heterogeneous telemetry variables. In multivariate aircraft telemetry, different sensor channels may respond to the same abnormal event with different temporal delays due to asynchronous sensing, transmission latency, or heterogeneous physical dynamics. Therefore, applying a single shared temporal response to all latent variables may be insufficient for capturing anomaly-sensitive temporal patterns.

**Figure 3:**
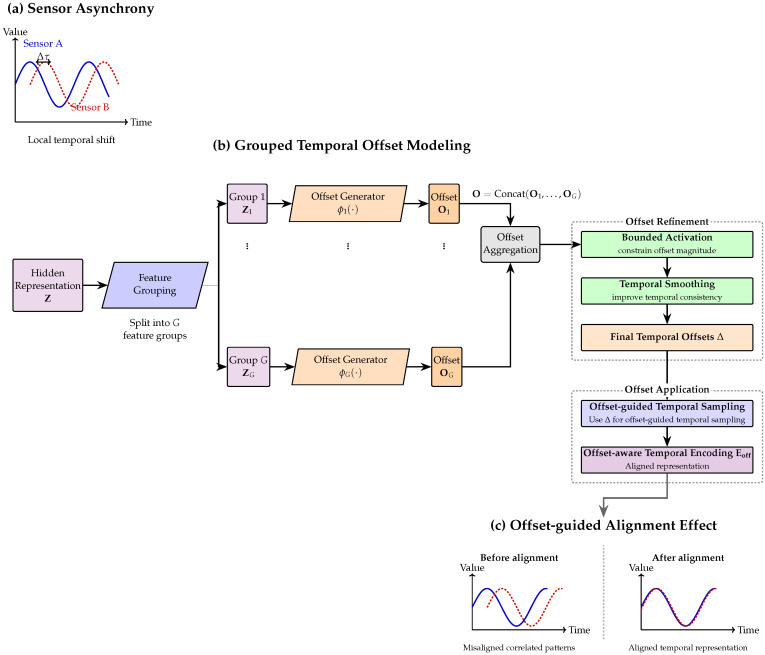
Mechanism of the grouped temporal offset modeling branch. (**a**) Correlated telemetry signals may exhibit asynchronous responses, leading to local temporal misalignment. (**b**) The hidden representation is partitioned into feature groups, and each group is processed by a dedicated offset generator to estimate group-wise temporal offsets. These offsets are aggregated and refined through bounded activation and temporal smoothing to obtain the final offsets Δ, which are then used for offset-guided temporal sampling and offset-aware temporal encoding. (**c**) The learned offsets help reduce temporal misalignment and yield a more coherent aligned representation.

As illustrated in Figure 3, given the normalized hidden representation

(7) $$Z\in R^{T\times d_{\mathrm{model}}},$$

where *T* denotes the temporal length, and $d_{\mathrm{model}}$ denotes the latent feature dimension, the feature channels are first partitioned into *G* groups:

(8) $$Z\to \{Z_{1},Z_{2},\ldots ,Z_{G}\},$$

where each group $Z_{g}\in R^{T\times d_{g}}$ represents a latent feature subspace and $\sum_{g=1}^{G}d_{g}=d_{\mathrm{model}}$. This grouping strategy allows the model to estimate different temporal displacement patterns within different feature subspaces, rather than imposing a single global offset across all latent variables.

For each feature group, a dedicated offset generator $\phi_{g}\left(\cdot \right)$ is used to estimate group-wise temporal offsets:

(9) $$O_{g}=\phi_{g}\left(Z_{g}\right),\;g=1,\ldots ,G.$$

Here, $O_{g}$ denotes the offset representation generated from the *g*-th latent feature group. The group-wise offsets are then aggregated to form a unified offset representation:

(10) $$O=\mathrm{Concat}(O_{1},O_{2},\ldots ,O_{G}).$$

This aggregation preserves the group-specific temporal information while providing a unified offset representation for subsequent temporal refinement.

To avoid unstable or excessively large temporal displacements, the aggregated offsets are passed through bounded activation and temporal smoothing. The resulting final temporal offsets are denoted as

(11) $$\Delta =S\left(\tanh(O)\right),$$

where tanh(·) constrains the offset magnitude, and S(·) denotes temporal smoothing. This refinement step encourages temporally coherent offset patterns and reduces abrupt offset changes across adjacent time steps.

The refined offsets Δ are then used for offset-guided temporal sampling. Instead of attending only to the original temporal positions, the model uses Δ to adjust the temporal sampling locations and produce an offset-aware temporal encoding:

(12) $$E_{\mathrm{off}}=\mathrm{Sample}\left(Z;\Delta \right).$$

In practice, this operation allows the temporal representation to incorporate locally shifted information and better align correlated but asynchronous telemetry patterns. The resulting offset-aware representation is then used in the subsequent attention computation for anomaly-sensitive feature learning.

Compared with a standard Transformer block, this mechanism introduces an explicit adaptive temporal-alignment operation. The offset generators estimate local temporal shifts from grouped latent features, while the refinement and sampling stages transform these shifts into aligned temporal representations. As a result, FG-DeformT can better accommodate heterogeneous temporal responses across latent feature groups, which is particularly important for flight-level anomaly detection under cross-flight distribution shifts.

### 4.4. Deformable Key-Value Temporal Sampling

Unlike an auxiliary branch that only provides an additional representation, the proposed offset mechanism is directly coupled with the attention computation by deforming the temporal sampling positions of the key and value representations.

Given the projected query, key, and value tensors:

(13) $$Q,K,V\in R^{H\times T\times d_{h}},$$

where $d_{h}=d_{\mathrm{model}}/H$, the offset $\Delta_{h,t}$ shifts the sampling position for the key and value sequences. For each head *h* and time step *t*, the deformed key and value are obtained by linear interpolation:

(14) $$K_{h,t}^{\prime }=\mathrm{Interp}\left(K_{h},t+\Delta_{h,t}\right),$$

(15) $$V_{h,t}^{\prime }=\mathrm{Interp}\left(V_{h},t+\Delta_{h,t}\right).$$

Since $t+\Delta_{h,t}$ may be non-integer, linear interpolation is adopted between the two nearest temporal positions. Boundary positions are clipped to the valid range [1,T]. This operation enables the model to attend to temporally shifted local patterns and thus capture asynchronous responses among heterogeneous telemetry variables.

The attention output is then computed using the original query and the deformed key-value representations:

(16) $$\mathrm{DeformAttn}\left(Q,K^{\prime },V^{\prime }\right)=\mathrm{softmax}\left(\frac{Q{K^{\prime }}^{⊤}}{\sqrt{d_{h}}}\right)V^{\prime }.$$

This formulation differs from standard self-attention, which uses K and V at their original temporal positions. It also differs from a generic multi-branch fusion module because the learned offsets explicitly determine the temporal sampling locations used in attention computation.

### 4.5. Transformer-Based Temporal Representation Learning

Each deformable temporal block consists of deformable multi-head attention, residual connections, layer normalization, and a feed-forward network. The block output is computed as

(17) $$Z^{\prime }=Z+\mathrm{DeformMHA}\left(Z\right),$$

(18) $$Z^{\mathrm{out}}=Z^{\prime }+\mathrm{FFN}\left(Z^{\prime }\right).$$

Through this design, the model preserves the global temporal modeling capability of the Transformer backbone while introducing adaptive temporal deformation for local asynchronous patterns. The grouped offset mechanism allows different latent feature subspaces or attention heads to learn different temporal displacements, which is useful for aircraft telemetry, where different physical processes may exhibit different time delays.

### 4.6. Classification Head and Training Objective

After the final deformable temporal block, the sequence representation is aggregated by average pooling:

(19) $$h=\frac{1}{T}\sum_{t=1}^{T}Z_{t}^{\left(L\right)},$$

where $Z^{\left(L\right)}$ denotes the output of the last block. The anomaly prediction is produced by a linear classifier:

(20) $$\hat{y}=\mathrm{Linear}\left(h\right).$$

For the primary anomaly detection task, the model is trained as a supervised binary classifier. For the downstream fault-isolation analysis, the output layer is adapted to the number of valid fault categories and optimized with a multi-class classification objective. The fault-isolation task is used as a boundary analysis to examine whether the learned representation is informative for fine-grained diagnosis, rather than as the primary target of the proposed framework.

## 5. Experimental Setup

### 5.1. Dataset Description

The main experiments are conducted on the ALFA (Air Lab Fault and Anomaly) dataset [21], a real-flight UAV Fault and anomaly-detection benchmark collected from autonomous fixed-wing flights. The dataset was recorded using a Carbon-Z T-28 fixed-wing UAV (E-flite/Horizon Hobby, Champaign, IL, USA) equipped with an onboard computer and auxiliary sensing modules. It contains normal flights as well as fault-injected flights, including engine failures and sudden control-surface faults such as rudder, elevator, and aileron-related anomalies.

In this study, ALFA is used for two related tasks. The first task is binary anomaly detection, where each window is classified as normal or anomalous. This task serves as the primary evaluation setting. The second task is downstream fault isolation, where anomalous windows are further classified into fault categories. This task is used as a boundary analysis to examine whether the learned representations remain informative for fine-grained fault-type discrimination.

After preprocessing and sliding-window construction, the binary anomaly detection task contains 16,004 windows from 33 flights. Among them, 2799 windows are normal, and 13,205 windows are anomalous. For the downstream fault-isolation task, the normal class is excluded, and only fault windows are retained. The reconstructed fault-isolation subset contains 5446 anomalous windows from 36 flight-level groups. The processed window-level statistics are summarized in Table 1.

For the downstream fault-isolation task, the valid fault categories are Engine, Elevator, Rudder, and Aileron. The corresponding class distribution is highly imbalanced, with 2194 Engine windows, 110 Elevator windows, 349 Rudder windows, and 2793 Aileron windows. This imbalance is considered when interpreting fault-isolation results, which are reported as a boundary analysis rather than as the main evidence of model effectiveness.

### 5.2. External Dataset: THOR

To evaluate the external applicability of the proposed framework, we conduct an additional validation experiment on the THOR telemetry dataset [22]. While ALFA serves as the primary in-domain benchmark for aircraft fault and anomaly detection, THOR is used as an out-of-dataset benchmark to assess cross-run generalization under a different telemetry source and task definition.

In the collected THOR subset, 15 H5 source files were initially scanned, among which 14 files were retained after excluding one file without the required gpsStatus field. Since each source file may contain multiple operational runs, the combination of source-file identity and run number is used as the grouping variable, resulting in 74 valid run-level groups. All windows from the same run are assigned to the same split to reduce leakage caused by highly correlated samples.

The THOR task is formulated as GPS-status anomaly detection. After leakage-safe preprocessing, 33,923 sliding-window samples are generated, with 13,417 positive windows and 20,506 negative windows. The final input contains 73 telemetry features after excluding direct label fields, target-proxy variables, temporal identifiers, and grouping identifiers, including label, gpsStatus, imuStatus, navValid, mode, time stamps, file identifiers, and run identifiers.

THOR is not intended to replicate the exact fault taxonomy of ALFA. Instead, it complements the ALFA experiments by providing an external, grouped-evaluation setting to test whether the proposed method remains applicable beyond the primary dataset. The statistics of the THOR subset are summarized in Table 2.  

### 5.3. Data Preprocessing

For ALFA, the raw telemetry data are organized by flight identity. For THOR, samples are organized by run-level identity, defined by the combination of source-file identity and run number. This grouping is performed before sliding-window construction to prevent correlated windows from the same flight or run from being assigned to different splits.

Within each flight or run, continuous multivariate telemetry sequences are converted into fixed-length samples using a sliding-window strategy. For an input sequence length of *T*, each sample is represented as $X\in R^{T\times D}$, where *D* denotes the number of input variables. Windows are generated only within the same flight or run and are not allowed to cross group boundaries. The same windowing procedure is applied to all compared models within each dataset.

For the ALFA binary anomaly detection task, window labels are assigned according to whether the corresponding segment belongs to a fault state. For the extended ALFA fault-isolation task, anomalous windows are further re-labeled into valid fault categories after excluding the normal class. For the THOR external validation task, labels are derived from GPS-status abnormality. To avoid trivial leakage, direct label fields, target-proxy variables, temporal identifiers, and grouping identifiers, including label, gpsStatus, imuStatus, navValid, mode, time stamps, file identifiers, and run identifiers, are excluded from the THOR input features.

Feature selection, missing-value handling, normalization, and window construction are performed consistently within each experimental setting. Normalization parameters are fitted only on the training split and then applied to the corresponding validation or test split. ALFA is used as the primary in-domain benchmark for aircraft anomaly detection and fault isolation, whereas THOR serves as an external telemetry benchmark for GPS-status anomaly detection under run-level grouped evaluation.

### 5.4. Validation Protocols

To evaluate model generalization under leakage-aware settings, we adopt 5-fold GroupKFold validation for both ALFA and THOR. For ALFA, the grouping unit is the flight identity, and all windows from the same flight are assigned to the same fold. For THOR, the grouping unit is defined by the combination of source-file identity and run number, and all windows from the same run are assigned to the same fold. This design prevents highly correlated windows from the same flight or run from appearing simultaneously in training and testing sets.

Grouped validation is used as the primary evaluation protocol. Under this setting, each validation fold consists of unseen flights or unseen runs, which better reflects practical deployment scenarios than random window-level splitting. All compared models are evaluated under the same grouped folds to ensure fair comparison.

Random window-level splitting is additionally reported only as a reference setting to illustrate the potential inflation caused by correlated sliding windows. In the random protocol, windows are randomly assigned to training and testing sets without considering flight or run identity. Although this setting is commonly used in time-series learning, it may yield optimistic results when adjacent or overlapping windows from the same trajectory appear in both the training and test sets. Therefore, the random protocol is not used as the primary basis for model comparison.

For the extended ALFA fault-isolation task, flight-level grouping is also preserved to maintain consistency with the leakage-aware evaluation principle. For the THOR external validation task, run-level grouping is used to assess cross-run generalization under a different telemetry source. Results are reported using Precision, Recall, F1-score, and AUROC where applicable, with mean and standard deviation computed over the five grouped folds.

### 5.5. Baseline Methods

To evaluate the proposed FG-DeformT, we compare it with representative temporal modeling baselines under the same leakage-aware grouped validation protocols. The compared methods include classical recurrent models, Transformer-based models, modern multivariate time-series models, and convolutional temporal baselines.

LSTM is included as a classical recurrent baseline because recurrent networks have been widely used for sequential fault diagnosis and aircraft telemetry modeling. A vanilla Transformer is used as a representative self-attention baseline to assess whether the proposed grouped deformable temporal mechanism provides additional benefits beyond standard global temporal attention. In addition, iTransformer and PatchTST are selected as recent multivariate time-series Transformer baselines due to their strong performance in sequence modeling tasks.

For the THOR external validation experiment, a CNN-1D baseline is also included. This model serves as a strong local temporal pattern extractor and provides a reference for evaluating whether deformable temporal modeling offers additional benefits over convolutional feature extraction in GPS-status anomaly detection. All baselines are trained and evaluated using the same preprocessing pipeline, input windows, and grouped validation protocol within each dataset.

For the extended ALFA fault-isolation analysis, we compare the vanilla Transformer with the full FG-DeformT to examine whether the proposed grouped temporal offset mechanism remains beneficial for fine-grained fault-type classification. This comparison is used as a boundary analysis to clarify the contribution and limitation of the grouped temporal offset mechanism, rather than as the main evidence for model superiority.

### 5.6. Implementation Details

All models are implemented in PyTorch (v2.5.1) with Python (v3.11.7). For both ALFA and THOR, continuous telemetry sequences are converted into fixed-length sliding-window samples after group-level organization. The final evaluation is conducted using a 5-fold GroupKFold validation. For ALFA, folds are constructed according to flight identity, whereas for THOR, folds are constructed according to run-level identity. The same folds are used for all compared models within each dataset to ensure fair comparison.

The proposed FG-DeformT uses a Transformer-style backbone with linear input embedding, learnable positional encoding, stacked deformable temporal blocks, and a linear classification head. Unless otherwise specified, model hyperparameters are kept fixed within each experimental setting. The models are optimized using the AdamW optimizer. For binary anomaly detection, the model is trained with a binary classification objective. For the extended fault-isolation task, the output layer is adapted to the number of valid fault categories and optimized using a multi-class classification objective.

To avoid evaluation leakage, normalization parameters are fitted only on the training folds and then applied to the corresponding validation folds. For imbalanced settings, class-weighting strategies are considered where appropriate, especially in the extended fault-isolation analysis. Model performance is evaluated using Precision, Recall, F1-score, and AUROC for binary anomaly detection. For fault isolation, Accuracy, Weighted F1, Macro F1, and per-class F1-scores are reported. All final results are reported as mean and standard deviation over the five grouped folds.

## 6. Experimental Results

### 6.1. Main Results on ALFA Under Random and Flight-Level Protocols

Table 3 compares the anomaly detection performance on ALFA under two validation protocols. The random window-level protocol is included as a reference to show the possible inflation caused by overlapping sliding windows, whereas the flight-level grouped protocol is used as the main evaluation setting for cross-flight generalization.

Under random window-level splitting, all compared models achieve near-saturated results, with F1-scores close to or above 0.98. FG-DeformT also reaches a near-perfect F1-score of 1.000 under the same protocol. This result should not be interpreted as evidence of genuine generalization. Rather, it indicates that random window-level evaluation can yield overly optimistic estimates when highly overlapping windows from the same flight trajectory are distributed across the training and test sets. Additional leakage diagnostics using shallow decision trees on window-level summary statistics further support this interpretation, showing that random splitting can exploit strongly correlated trajectory-specific patterns.

When the evaluation is changed to flight-level grouped validation, the performance of the baseline models drops substantially. The F1-score of LSTM decreases from 0.978 to 0.616, and that of the vanilla Transformer decreases from 0.989 to 0.623. Recent multivariate time-series models show even stronger degradation, with iTransformer and PatchTST obtaining F1-scores of 0.231 and 0.311, respectively. These results confirm that flight-level evaluation poses a much stricter generalization challenge than random window-level splitting.

In contrast, FG-DeformT maintains strong performance under flight-level validation, achieving a Precision of 0.925, a Recall of 0.941, and an F1-score of 0.933. Compared with the baseline models, FG-DeformT shows a more stable ability to detect anomalous patterns in unseen flights. This supports the use of grouped, deformable temporal modeling for the primary binary anomaly-detection task under leakage-aware cross-flight evaluation.

**Figure 4:**
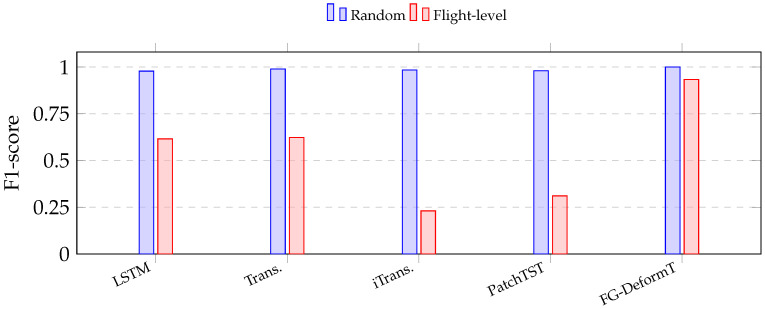
F1-score comparison on ALFA under random window-level and flight-level grouped validation protocols. Detailed numerical values are reported in Table 3. Random-split results are included only as a reference, while flight-level grouped validation is used as the primary evaluation protocol.

Figure 4 visualizes the F1-score gap between the two protocols. The near-saturated random-split results across different model families indicate that the inflation is systematic rather than model-specific. By contrast, the flight-level results better reveal the difficulty of generalizing to unseen flights. Under this stricter setting, FG-DeformT achieves the highest F1-score among the compared methods, suggesting that the proposed temporal deformation mechanism improves robustness when flight-level training–testing overlap is removed.

Overall, these findings show that random window-level results should be treated only as a diagnostic reference. The flight-level grouped protocol provides a more reliable basis for evaluating aircraft anomaly detection models, and the remaining analyses therefore focus on leakage-aware grouped validation.

### 6.2. External Validation on THOR

To further examine whether the proposed framework remains applicable beyond ALFA, we conduct an external validation experiment on the THOR dataset under the run-level grouped protocol. Unlike ALFA, which focuses on aircraft anomaly detection and downstream fault isolation, THOR is used here for GPS-status anomaly detection under a different telemetry source and task definition. Therefore, this experiment is intended to assess external applicability and cross-run generalization rather than to replicate the exact ALFA fault taxonomy.

Table 4 reports the results on THOR. Under the leakage-aware run-level grouped protocol, FG-DeformT achieves an F1-score of 0.8010±0.0413 and an AUROC of 0.8860±0.0174. These results indicate that FG-DeformT remains competitive under an external telemetry setting, although its advantage is not uniform across all metrics. Compared with the CNN-1D baseline, FG-DeformT obtains a slightly higher Recall and AUROC, whereas CNN-1D achieves a slightly higher Accuracy, Precision, and F1-score.

This result suggests that the benefit of deformable temporal modeling is task-dependent. For the THOR GPS-status anomaly detection task, local convolutional temporal patterns already provide a strong baseline. Nevertheless, FG-DeformT shows competitive ranking ability and sensitivity to abnormal windows, supporting its broader applicability under leakage-aware grouped evaluation. Accordingly, the THOR experiment is interpreted as an external applicability analysis rather than evidence of universal superiority across all telemetry tasks.

### 6.3. Boundary Analysis on Downstream Fault Isolation

In addition to the primary binary anomaly detection task, we further conduct a downstream fault-isolation analysis to examine the applicability boundary of FG-DeformT. In this experiment, only fault windows are retained and categorized into four fault types: Engine, Elevator, Rudder, and Aileron. The reconstructed fault-isolation subset contains 5446 windows from 36 flight-level groups, as summarized in Table 5. The class distribution is highly imbalanced, with Aileron and Engine dominating the subset, while Elevator contains only 110 windows.

Table 6 reports the fault-isolation results under flight-level grouped evaluation. Overall, the results show a mixed pattern. FG-DeformT achieves slightly higher Accuracy and Weighted F1 than the vanilla Transformer, increasing Accuracy from 0.779 to 0.784 and Weighted F1 from 0.794 to 0.803. It also improves the F1-score for Engine and Aileron faults. However, the vanilla Transformer obtains a slightly higher Macro F1 and better Rudder F1. Both models fail to recognize Elevator faults in the global evaluation.

These results suggest that the grouped temporal offset mechanism does not uniformly improve fine-grained fault-type classification. Its benefit is more evident for the primary binary anomaly detection task and for selected fault categories, but it is less stable when the task requires distinguishing among highly imbalanced fault types. The poor Elevator performance is mainly associated with the extremely limited number of Elevator samples and the uneven class coverage across flight-level folds.

Therefore, the downstream fault-isolation experiment is interpreted as a boundary analysis rather than as the main evidence for model effectiveness. The results indicate that FG-DeformT retains useful diagnostic information for some fault types, but fine-grained multi-fault diagnosis likely requires additional task-specific mechanisms, such as class-balanced representation learning, fault-discriminative constraints, or more balanced multi-fault flight data.

## 7. Ablation Study and Offset Analysis

To further clarify the contribution of the temporal offset mechanism, we conduct a controlled ablation study on the ALFA binary anomaly detection task under the flight-level grouped validation protocol. The ablated variant is denoted as FG-DeformT without temporal offset. In this variant, all learned temporal offsets are forced to zero, so that the deformable sampling positions collapse to the original temporal indices. The remaining Transformer-based backbone, training objective, and grouped validation setting are kept unchanged. This design allows us to isolate the effect of adaptive temporal deformation while keeping the rest of the model architecture comparable.

As shown in Table 7, the no-offset variant remains competitive, achieving an average Accuracy of 0.905±0.104 and an average F1-score of 0.935±0.069 in the controlled offset diagnostic runs. This result indicates that the Transformer-based temporal backbone itself already provides strong anomaly-discriminative representations under the flight-level grouped validation protocol. Nevertheless, under the same diagnostic setting, the full FG-DeformT improves the average Accuracy to 0.952±0.086 and the average F1-score to 0.965±0.050. The lower standard deviation of the full model further suggests that the temporal offset mechanism contributes to more stable cross-flight generalization.

It should be noted that Table 7 serves as a controlled ablation within the FG-DeformT family, whereas Table 3 reports the main comparison with external baselines. Therefore, the results in this section are used to assess the relative effect of disabling temporal offsets under the same diagnostic setting, rather than to replace the main baseline comparison results.

The offset statistics further support this interpretation. In the full FG-DeformT, the validation offset debug output shows non-zero offsets in both deformable layers, indicating that the model actively learns temporal displacement patterns. By contrast, in the no-offset variant, the offsets are exactly zero across all heads and layers during validation, confirming that the ablation effectively removes the temporal deformation operation. Therefore, the performance difference in Table 7 can be attributed to the presence or absence of adaptive temporal deformation rather than to changes in the training protocol or backbone architecture.

We quantify this effect using the local peak alignment error, defined as the absolute difference between the peak positions of the two signals within the selected local segment. In the illustrated example, the peak alignment error is reduced from 12 time steps before alignment to 0 time steps after alignment. This visualization supports the interpretation that the learned temporal offsets can compensate for local timing mismatch and provide a more coherent temporal representation. This analysis is intended as an interpretable diagnostic example rather than a replacement for the quantitative ablation results reported in Table 7.

These findings suggest that the grouped temporal offset branch should not be viewed merely as an additional parameterized component. Instead, it provides an explicit adaptive temporal-alignment mechanism by deforming the sampling positions of key and value representations in attention computation. This mechanism is consistent with the asynchronous telemetry motivation of this study: different sensor variables may exhibit local temporal shifts, and the model benefits from a learnable mechanism for adjusting temporal sampling positions.

**Figure 5:**
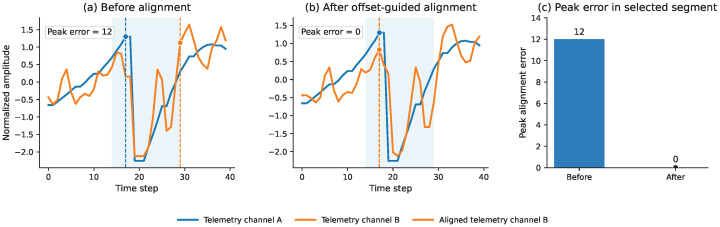
Offset-guided temporal alignment analysis based on a representative anomalous validation window from the ALFA offset diagnostic run. (**a**) Before alignment, two correlated telemetry channels exhibit a visible local temporal mismatch, and their dominant local peaks occur at different time steps. (**b**) After offset-guided temporal sampling, the local peak positions become more coherent. (**c**) The local peak alignment error in the selected segment is reduced after applying the learned temporal offsets. Dashed vertical lines indicate the detected local peak positions used to compute the peak alignment error.

To further interpret the learned temporal offsets, Figure 5 provides an offset-guided alignment visualization based on a representative anomalous validation window from the ALFA offset diagnostic run. Two correlated telemetry channels are selected to illustrate the local temporal mismatch. Before alignment, their dominant local peaks occur at different time steps, indicating a clear local timing discrepancy. After applying the learned offset-guided temporal sampling, the peak positions become more coherent.

Overall, the ablation results show that the temporal offset mechanism improves the average performance and stability of FG-DeformT under flight-level grouped validation. At the same time, the competitive performance of the no-offset variant indicates that the Transformer-based backbone also plays an important role. Thus, the contribution of the proposed method should be understood as the combination of a strong temporal backbone and an offset-aware temporal deformation mechanism for modeling heterogeneous aircraft telemetry dynamics.

## 8. Discussion

The experimental results highlight the importance of evaluating aircraft telemetry anomaly detection under leakage-aware protocols. In random window-level splitting, highly correlated sliding windows from the same flight can appear in both the training and testing sets, leading to overly optimistic performance estimates. By contrast, the flight-level grouped protocol evaluates whether a model can generalize to unseen flights, which is closer to practical deployment. The sharp performance gap between random and flight-level evaluation, therefore, suggests that cross-flight generalization should be treated as a central requirement for safety-critical telemetry modeling rather than as a secondary validation setting.

Under this stricter evaluation protocol, FG-DeformT demonstrates strong performance on the primary binary anomaly detection task. The grouped temporal offset mechanism is designed to address the local temporal misalignment that may arise from asynchronous sensing, transmission latency, and heterogeneous physical responses across telemetry variables. By estimating group-specific offsets and using them for offset-guided temporal sampling, the model introduces an explicit temporal-alignment mechanism into the Transformer-based representation learning process. The offset analysis further confirms that the full model learns non-zero temporal offsets, whereas the no-offset variant removes this temporal deformation operation. This supports the interpretation that the proposed branch is not merely an additional parameterized component, but an explicit mechanism for modeling asynchronous temporal dynamics.

At the same time, the ablation results suggest that the benefit of the temporal offset branch should be interpreted carefully. The no-offset variant remains competitive, indicating that the Transformer-based backbone and the flight-level training protocol already provide strong representations for discriminating anomalies. However, the full FG-DeformT achieves higher average accuracy and lower fold-level variability, suggesting that the offset mechanism contributes to more stable cross-flight generalization. Therefore, the contribution of FG-DeformT should be understood as the combination of a strong temporal backbone and an offset-aware alignment mechanism, rather than as a claim that temporal offsets uniformly dominate all metrics in every fold.

The downstream fault-isolation results further clarify the applicability boundary of the current design. While FG-DeformT is effective for binary anomaly detection, fine-grained fault-type classification remains substantially more challenging. The reconstructed fault-isolation subset is highly imbalanced, with very limited samples for certain categories, such as Elevator. Under flight-level grouped validation, such an imbalance becomes more severe because some folds may contain limited or uneven coverage of minority fault types. As a result, the fault-isolation experiment is better interpreted as a boundary analysis rather than as the main evidence for model superiority. The mixed results indicate that group-specific temporal deformation is useful for anomaly-sensitive representation learning, but additional task-specific mechanisms may be required for robust multi-class fault diagnosis.

The THOR external validation experiment provides complementary evidence regarding the broader applicability of the proposed framework. Unlike ALFA, THOR is used as an out-of-dataset telemetry benchmark with a different task definition and grouped run-level evaluation. The results suggest that the proposed framework remains applicable under a different telemetry source and a leakage-aware grouped setting. However, this external validation should not be over-interpreted as evidence of full domain generalization across aircraft platforms or anomaly definitions. Instead, it serves as an additional robustness check showing that the framework can be transferred to a different telemetry dataset when appropriate grouped evaluation and leakage-safe preprocessing are adopted.

Several limitations remain. First, the available datasets are still limited in scale and diversity compared with the variability of real-world aircraft operations. More flight scenarios, aircraft platforms, environmental conditions, and fault modes are needed to fully evaluate deployment robustness. In particular, more detailed modeling of specific aviation failure signatures, such as attitude-sensor drift, abrupt attitude changes, engine-temperature anomalies, and vibration-related patterns, will require richer sensor modalities and more fine-grained fault annotations in future datasets. Second, the current fault-isolation analysis is affected by severe class imbalance, especially for minority fault categories. Future work should incorporate class-balanced representation learning, fault-discriminative objectives, or data augmentation strategies tailored to rare fault types. Third, although the grouped temporal offset mechanism improves interpretability by exposing learned temporal displacements, the physical meaning of specific offsets still requires further domain-level validation. Future work may also incorporate graph-structured temporal modeling to explicitly represent physical or statistical dependencies among aircraft sensor variables, which may further improve the modeling of multivariate coupling in complex aviation systems. Finally, real-time deployment would require additional assessment of computational latency, threshold stability, and robustness under streaming telemetry conditions.

Overall, these findings suggest that leakage-aware flight-level evaluation is essential for aircraft telemetry anomaly detection. FG-DeformT provides a practical and interpretable framework for binary anomaly detection under cross-flight distribution shifts, while external validation and fault-isolation analyses clarify its robustness and current limitations.

## 9. Conclusions

This study investigated aircraft telemetry anomaly detection under leakage-aware flight-level evaluation. We showed that random window-level splitting can overestimate model performance because highly correlated windows from the same flight may appear in both training and testing sets. To provide a stricter assessment of cross-flight generalization, we adopted a flight-level grouped validation protocol in which each validation fold contains unseen flights.

Under this setting, we proposed FG-DeformT, a grouped deformable Transformer that combines Transformer-based temporal modeling with group-specific temporal offset generation. By learning offset-aware temporal sampling positions, FG-DeformT provides an explicit mechanism for modeling local temporal misalignment among heterogeneous telemetry variables.

Experiments on ALFA demonstrate that flight-level evaluation is substantially more challenging than random window-level evaluation. FG-DeformT maintains strong performance on the primary binary anomaly detection task, while the ablation study and offset-guided visualization further indicate that the temporal offset mechanism improves model stability and provides interpretable evidence of local temporal alignment. External validation on THOR suggests that the framework remains applicable across different telemetry sources and grouped evaluation settings. Meanwhile, the downstream fault-isolation analysis shows that fine-grained multi-fault diagnosis remains sensitive to class imbalance and category-specific data coverage.

Overall, this study highlights the importance of leakage-aware flight-level evaluation for safety-critical telemetry modeling. The results suggest that group-specific temporal deformation is a practical and interpretable direction for aircraft anomaly detection under cross-flight distribution shifts. Future work will focus on broader flight scenarios and fault modes, class-balanced fault isolation, domain-level interpretation of learned offsets, graph-structured sensor modeling, and real-time deployment under streaming telemetry conditions.

## Figures and Tables

**Table 1:** Window-level statistics of the processed ALFA dataset.

Task	Split Unit	Total Windows	Normal	Anomalous
Binary anomaly detection	Flight	16,004	2799	13,205
Fault isolation	Fault window	5446	–	5446

**Table 2:** Statistics of the THOR external validation subset after leakage-safe preprocessing.

Item	Value
Scanned H5 source files	15
Usable source files	14
Excluded files	1 without gpsStatus
Run-level groups	74
Input feature dimension	73
Total windows	33,923
Positive windows	13,417
Negative windows	20,506
Positive ratio	0.3955
External validation task	GPS-status anomaly detection
Evaluation protocol	Run-level grouped split

**Table 3:** Anomaly detection performance on ALFA under random window-level and flight-level grouped validation protocols. Random-split results are reported only as a reference for illustrating performance inflation caused by window-level random evaluation; flight-level grouped validation is used as the primary protocol for model comparison.

Method	Protocol	Precision	Recall	F1-Score
Classical Baselines				
LSTM [23]	Random	0.982	0.975	0.978
LSTM [23]	Flight-level	0.654	0.582	0.616
Transformer Baseline				
Transformer [24]	Random	0.991	0.988	0.989
Transformer [24]	Flight-level	0.642	0.605	0.623
Recent Multivariate Time-Series Models				
iTransformer [25]	Random	0.985	0.983	0.984
iTransformer [25]	Flight-level	0.301	0.190	0.231
PatchTST [26]	Random	0.981	0.979	0.980
PatchTST [26]	Flight-level	0.358	0.275	0.311
Proposed Method				
FG-DeformT (Ours)	Random	1.000	1.000	1.000
FG-DeformT (Ours)	Flight-level	0.925	0.941	0.933

**Table 4:** External validation results on the THOR dataset under run-level grouped evaluation. Results are reported as mean ± standard deviation over five grouped folds.

Method	Accuracy	Precision	Recall	F1-Score	AUROC
CNN-1D baseline	0.8583±0.0280	0.8242±0.0913	0.8070±0.0545	0.8127±0.0551	0.8849±0.0385
FG-DeformT	0.8423±0.0360	0.8069±0.1168	0.8156±0.0931	0.8010±0.0413	0.8860±0.0174

**Table 5:** Class distribution of the reconstructed ALFA fault-isolation subset.

Fault Type	Label Index	Number of Windows
Engine	0	2194
Elevator	1	110
Rudder	2	349
Aileron	3	2793
Total	–	5446

**Table 6:** Boundary analysis on downstream fault isolation under flight-level grouped evaluation. Per-class columns report F1-scores.

Method	Acc.	Weighted F1	Macro F1	Engine	Elevator	Rudder	Aileron
Vanilla Transformer	0.779	0.794	0.480	0.959	0.000	0.188	0.772
FG-DeformT	0.784	0.803	0.473	0.984	0.000	0.130	0.777

**Table 7:** Ablation study of the temporal offset mechanism under flight-level grouped validation on ALFA binary anomaly detection. Results are reported as mean ± standard deviation over five grouped folds from controlled offset diagnostic runs. The table compares the full FG-DeformT with its no-offset variant under the same diagnostic setting, while the main comparison with external baselines is reported in Table 3.

Variant	Accuracy	F1-Score
FG-DeformT without temporal offset	0.905±0.104	0.935±0.069
FG-DeformT	0.952±0.086	0.965±0.050

## Data Availability

The ALFA dataset analyzed in this study is publicly available from the original dataset publication. The THOR telemetry data used for external validation are available from the corresponding public data source cited in this study. The processed data and code generated during the current study are available from the corresponding author upon reasonable request.
